# Clinical Significance of Tear Matrix Metalloproteinase-9 Reaction Intensity in Thyroid Eye Disease

**DOI:** 10.3390/jcm15197671

**Published:** 2026-10-03

**Authors:** Do Hee Jung, Ji Ho Park, Jung Hyo Ahn, Ji Eun Lee

**Affiliations:** 1 Department of Ophthalmology, Pusan National University Yangsan Hospital, Pusan National University School of Medicine, Yangsan 50612, Republic of Korea; jdh414@naver.com (D.H.J.); jihopark529@gmail.com (J.H.P.); 2 Research Institute for Convergence of Biomedical Science and Technology, Pusan National University Yangsan Hospital, Yangsan 50612, Republic of Korea

**Keywords:** thyroid eye disease, matrix metalloproteinase-9, InflammaDry, clinical activity score, ocular surface inflammation

## Abstract

**Background/Objectives:** The objectives of this study were to investigate the association between tear matrix metalloproteinase-9 (MMP-9) grade and thyroid eye disease (TED) activity and to evaluate its discriminatory performance for active TED. **Methods:** This retrospective cross-sectional study included 322 patients with TED, including 188 with inactive disease and 134 with active disease. Tear MMP-9 reaction intensity was graded from 0 to 3 using InflammaDry, and active TED was defined as a Clinical Activity Score (CAS) of ≥3. Receiver operating characteristic analysis was used to assess discriminatory performance. Multivariable logistic regression was used to evaluate the independent association between a grade 3 result and active TED, adjusting for age, sex, and serum thyroid-stimulating hormone receptor antibody level. The main outcome measures were the association of tear MMP-9 grade with CAS, area under the receiver operating characteristic curve, adjusted odds ratio, and diagnostic performance for active TED. **Results:** Tear MMP-9 grade was significantly higher in active than in inactive TED and correlated positively with disease activity (Spearman’s ρ = 0.253, *p* < 0.001). Its overall discriminatory performance for active TED was modest (area under the curve, 0.619; 95% confidence interval, 0.552–0.682). A grade 3 result was independently associated with active TED (adjusted odds ratio, 8.65; 95% confidence interval, 2.86–26.17; *p* < 0.001) and demonstrated high specificity (97.9%) but low sensitivity (19.4%), with a positive predictive value of 86.7% and a positive likelihood ratio of 9.12. **Conclusions:** Tear MMP-9 grade was associated with TED activity and may provide clinically relevant information regarding ocular surface inflammation in TED.

## 1. Introduction

Thyroid eye disease (TED) is an autoimmune inflammatory disorder of the orbital soft tissues that can present with a broad spectrum of ocular manifestations, ranging from mild discomfort to vision-threatening complications. Because anti-inflammatory and immunomodulatory treatments are primarily indicated during the active phase, accurate assessment of disease activity is essential for treatment selection and optimal timing of intervention [1,2].

The Clinical Activity Score (CAS) is the most widely used clinical tool for assessing inflammatory activity in TED. It comprises seven inflammatory signs, and a score of ≥3 is commonly used to define active disease [1,3]. However, because CAS is based on clinical findings, its reproducibility may be affected by interobserver variability, and patients with similar scores may have different levels of underlying inflammatory activity [4]. These limitations have prompted interest in objective biomarkers that could complement conventional clinical assessment.

Ocular surface disease is common in TED and may result from both anatomical exposure and inflammatory alterations of the ocular surface [5,6]. Matrix metalloproteinase-9 (MMP-9) is a pro-inflammatory enzyme involved in extracellular matrix remodeling and ocular surface inflammation and is elevated in several inflammatory ocular surface disorders [7,8]. Tear MMP-9 positivity has been associated with active TED, suggesting that inflammatory tear biomarkers may provide additional objective information on disease activity [9]. To characterize tear MMP-9 expression beyond a dichotomous result, semiquantitative grading based on the intensity of the InflammaDry test line has been evaluated in dry eye disease [10,11,12].

Although tear MMP-9 grading has been investigated in ocular surface disease, its association with TED activity has not been fully established. Accordingly, we investigated the association between tear MMP-9 grade and TED activity and evaluated its ability to identify active TED.

## 2. Materials and Methods

### 2.1. Study Design and Patients

This retrospective study was conducted at the Department of Ophthalmology, Pusan National University Yangsan Hospital, Yangsan, Republic of Korea, between January 2018 and March 2026. The study was approved by the Institutional Review Board of Pusan National University Yangsan Hospital (IRB No. 55-2026-133; approval date: 13 August 2026) and was conducted in accordance with the tenets of the Declaration of Helsinki. The requirement for informed consent was waived by the Institutional Review Board because of the retrospective nature of the study. Patients diagnosed with TED who underwent CAS assessment and tear MMP-9 immunoassay during their initial visit were included. Patients with previous ocular surgery or ocular trauma, those with active ocular infection or non-TED-related ocular inflammation, those wearing contact lenses, those with Sjögren syndrome or other systemic diseases associated with ocular surface disease, those receiving topical corticosteroids or other topical anti-inflammatory medications, and pregnant or lactating women were excluded.

### 2.2. Assessment of TED Activity

TED activity was assessed using the 7-point CAS recommended by the European Group on Graves’ Orbitopathy [1,3]. The CAS consists of seven items, each scored as present or absent: spontaneous retrobulbar pain, pain on attempted upward or downward gaze, eyelid redness, conjunctival redness, eyelid swelling, inflammation of the caruncle and/or plica, and conjunctival edema. The total score was calculated as the sum of the seven items, with a maximum score of 7, and active TED was defined as a CAS of ≥3. CAS was assessed at the patient level rather than separately for each eye. A clinical sign present in at least one eye was counted as one point. Before Glandy CAS was introduced in 2025, the seven-item CAS was consistently assessed by one examiner (J.H.A.) in 255 patients; thereafter, the total seven-item CAS was calculated using Glandy CAS in 67 patients [13,14].

### 2.3. Tear MMP-9 Immunoassay and Ocular Surface Evaluation

**Figure 1:**
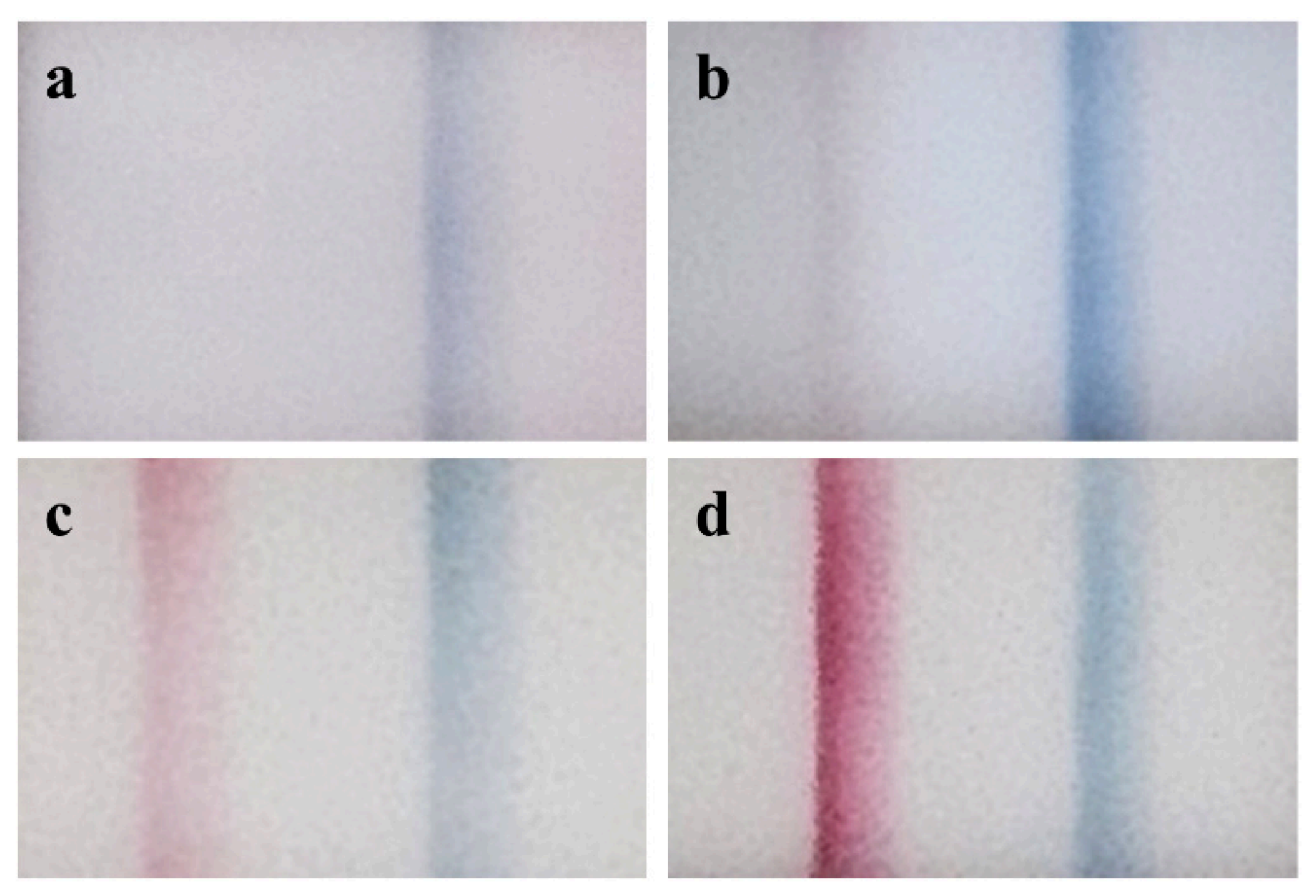
Representative InflammaDry immunoassay results according to tear matrix metalloproteinase-9 grade. (**a**) Grade 0, (**b**) grade 1, (**c**) grade 2, and (**d**) grade 3.

Tear MMP-9 was evaluated using a point-of-care immunoassay (InflammaDry; Quidel Corporation, San Diego, CA, USA) according to the established sampling procedure [15,16]. During routine testing, tear samples were collected without topical anesthesia by gently contacting the sample collector with several areas of the lower palpebral conjunctiva. The collector was then attached to the test cassette, and the absorbent tip was placed in the buffer solution after 5 s. To standardize the assessment of test-line intensity, the InflammaDry result was photographed 20 min after test initiation using a slit-lamp biomicroscope equipped with a digital single-lens reflex camera (D200; Nikon, Melville, NY, USA). Tear MMP-9 grading was performed by one examiner (J.E.L.). Each reagent band was graded at the time of testing using representative reference images: grade 0, negative; grade 1, weak positive; grade 2, moderate positive; and grade 3, strong positive (Figure 1) [10,11,12]. Subjective symptoms were assessed using the Ocular Surface Disease Index (OSDI), which ranges from 0 to 100, with higher scores indicating greater symptom-related disability [17]. Tear break-up time (TBUT) and corneal fluorescein staining (CFS) were evaluated by slit-lamp examination. TBUT was measured as the interval between the last complete blink and the first appearance of a dry spot after fluorescein instillation. CFS was graded using the Oxford grading scheme [18]. The Schirmer I test was performed without topical anesthesia using sterile Schirmer strips (Eagle Vision, Memphis, TN, USA). The strips were placed in the lateral third of the lower conjunctival fornix for 5 min, and the wetted length was measured in millimeters. Lipid layer thickness (LLT) and meiboscore were assessed using the LipiView ocular surface interferometer (TearScience Inc., Morrisville, NC, USA). Meiboscore was graded separately for the upper and lower eyelids as follows: grade 0, no meibomian gland loss; grade 1, loss of less than one-third of the total gland area; grade 2, loss of one-third to two-thirds; and grade 3, loss of more than two-thirds [19]. Except for tear MMP-9 testing, all ocular surface examinations were performed by a single examiner. The upper- and lower-eyelid meiboscores were summed for analysis. Clinical data from the right eye of each participant were used for analysis.

### 2.4. Statistical Analysis

Differences between the inactive and active TED groups were analyzed using the Mann–Whitney U test and Pearson’s chi-square test. Spearman’s rank correlation coefficient was used to assess the correlation between tear MMP-9 grade and CAS. Receiver operating characteristic (ROC) curve analysis was used to evaluate the discriminatory performance of ocular surface parameters, and areas under the curve (AUCs) were compared using the DeLong method [20]. Multivariable logistic regression analysis adjusted for age, sex, and serum TSH receptor antibody (TSHR-Ab) level was performed to assess the association between tear MMP-9 grade and active TED. Sensitivity, specificity, positive predictive value, negative predictive value, and positive and negative likelihood ratios were calculated for the tear MMP-9 cutoffs. Statistical analyses were performed using IBM SPSS Statistics for Windows (version 28.0; IBM Corp., Armonk, NY, USA). A two-sided *p* value < 0.05 was considered statistically significant.

## 3. Results

This retrospective study included 322 patients with TED, including 188 patients (58.4%) with inactive TED and 134 patients (41.6%) with active TED according to CAS. During the study period, 338 patients with TED underwent routine tear MMP-9 testing at their initial visit. A total of 16 patients were excluded because of previous ocular surgery (*n* = 11), contact lens wear (*n* = 1), Sjögren syndrome (*n* = 2), or topical corticosteroid use (*n* = 2), leaving 322 patients in the final analysis. No significant differences were observed in age or sex between the inactive and active TED groups. Corticosteroid treatment and orbital radiotherapy during subsequent follow-up were significantly more common in patients with active TED than in those with inactive TED. Serum TSH receptor antibody (TSHR-Ab) levels were also significantly higher in patients with active TED (Table 1).

Tear MMP-9 grades were significantly higher in patients with active TED than in those with inactive TED. No significant differences were observed in OSDI score, Schirmer I test results, meiboscore, or LLT between the two groups, whereas TBUT was shorter and CFS score was higher in patients with active TED; both differences were statistically significant (Table 2).

**Figure 2:**
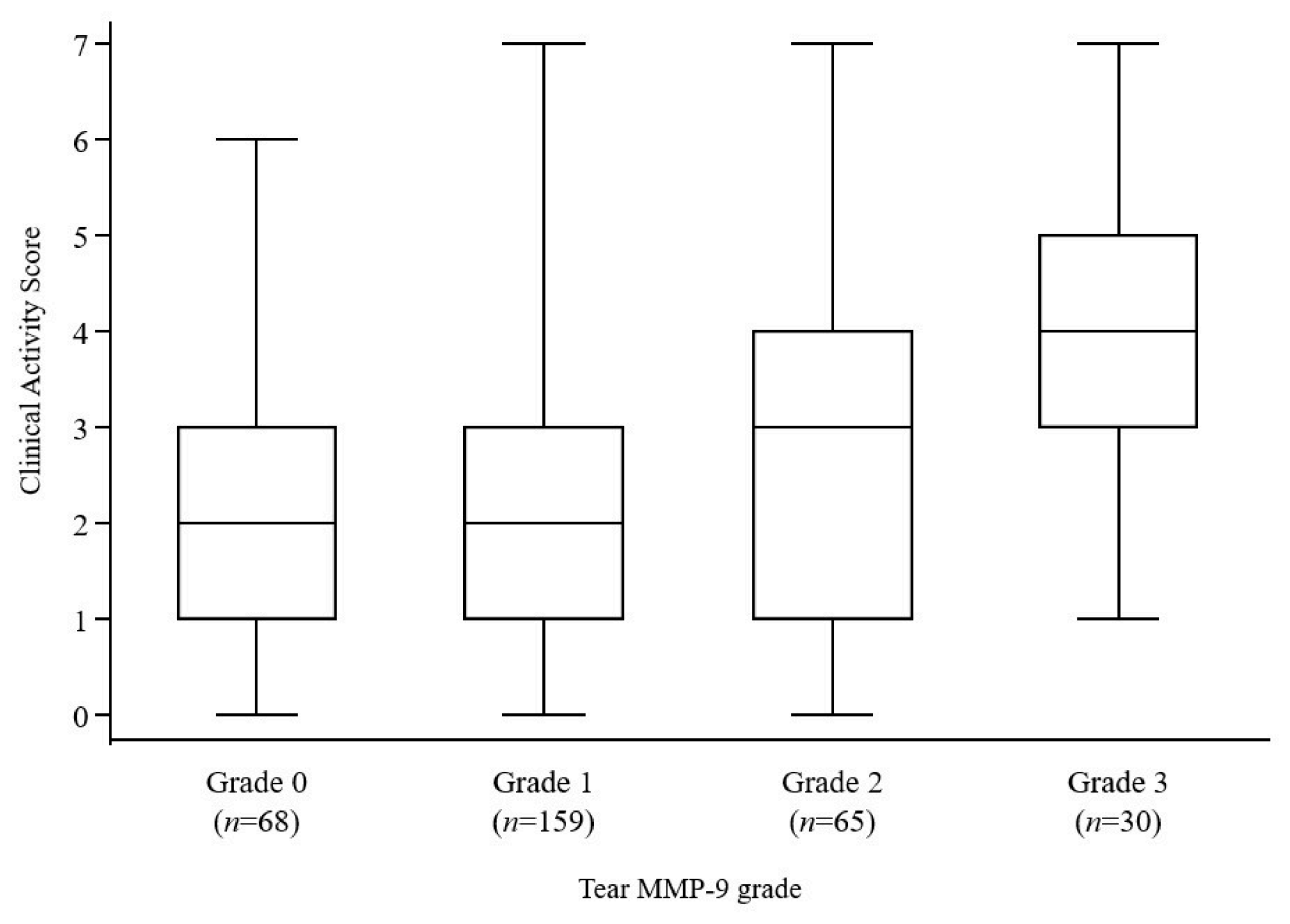
Distribution of Clinical Activity Score according to tear matrix metalloproteinase-9 grade. The boxes represent the interquartile range (IQR), the horizontal lines indicate the median, and the whiskers extend to the most extreme values within 1.5 × IQR. Clinical Activity Score increased with increasing tear matrix metalloproteinase-9 grade (Spearman’s ρ = 0.253; *p* < 0.001).

The median CAS was 2 (interquartile range [IQR], 1–3) for tear MMP-9 grades 0 and 1; 3 (IQR, 1–4) for grade 2; and 4 (IQR, 3–5) for grade 3. Tear MMP-9 grade was positively correlated with CAS (Spearman’s ρ = 0.253, *p* < 0.001) (Figure 2).

**Figure 3:**
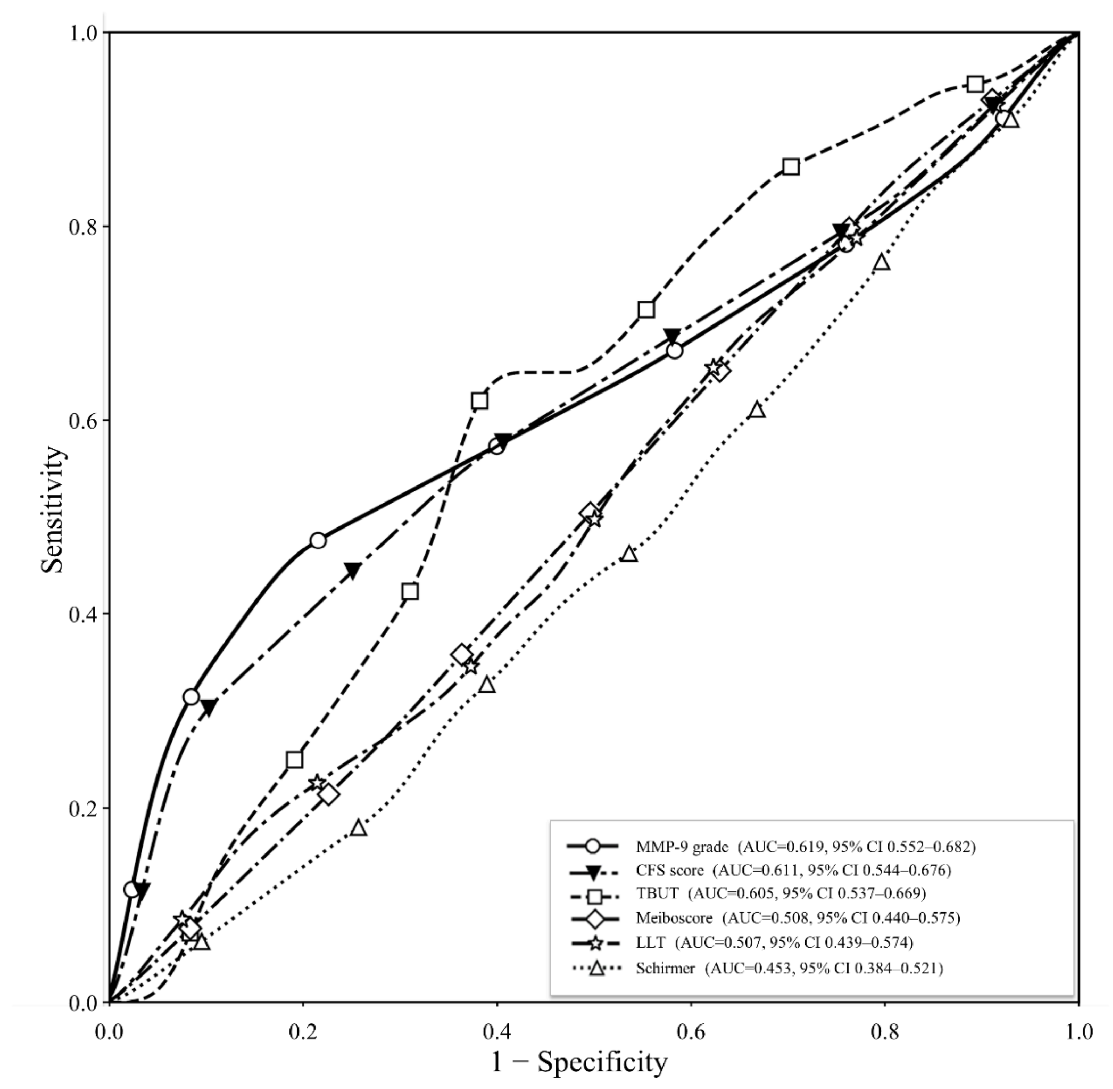
Receiver operating characteristic curve of tear matrix metalloproteinase-9 grade for identifying active thyroid eye disease.

The AUCs for identifying active TED were 0.619 (95% confidence interval [CI], 0.552–0.682) for tear MMP-9 grade, 0.611 (95% CI, 0.544–0.676) for CFS, 0.605 (95% CI, 0.537–0.669) for TBUT, 0.508 (95% CI, 0.440–0.575) for meiboscore, 0.507 (95% CI, 0.439–0.574) for LLT, and 0.453 (95% CI, 0.384–0.521) for the Schirmer I test. The AUC of tear MMP-9 grade was significantly greater than those of the Schirmer I test, meiboscore, and LLT, whereas no significant difference was observed between tear MMP-9 grade and TBUT (Figure 3).

Active TED was present in 63.2% of patients with tear MMP-9 grades 2–3 and in 32.6% of those with grades 0–1. Compared with grades 0–1, grades 2–3 were significantly associated with active TED after adjustment for age, sex, and serum TSHR-Ab level (adjusted odds ratio [OR], 3.42; 95% CI, 1.99–5.89; *p* < 0.001). Among patients with grade 3 tear MMP-9, 86.7% had active TED, compared with 37.0% of those with grades 0–2. Grade 3 tear MMP-9 remained independently associated with active TED after adjustment for age, sex, and serum TSHR-Ab level (adjusted OR, 8.65; 95% CI, 2.86–26.17; *p* < 0.001) (Table 3). Grade 3 tear MMP-9 demonstrated high specificity (97.9%) and a positive predictive value of 86.7%, with a positive likelihood ratio of 9.12. The grades 2–3 cutoff showed greater sensitivity than the grade 3 cutoff (44.8% vs. 19.4%) but lower specificity (81.4% vs. 97.9%) (Table 4).

## 4. Discussion

In this study, semiquantitatively assessed tear MMP-9 grade was significantly associated with TED activity, and CAS increased progressively with higher tear MMP-9 grades. Grade 3 showed the strongest association with active TED. Whereas previous studies either interpreted tear MMP-9 results dichotomously or evaluated tear MMP-9 reaction intensity in patients with dry eye disease, our findings indicate that graded interpretation of InflammaDry may provide additional clinical information on ocular surface status accompanying active TED [10,11,12,21,22,23].

In the present study, higher TSHR-Ab levels were observed in the active TED group, consistent with previous reports demonstrating an association between TSHR-Ab levels and Graves’ orbitopathy activity [24]. Systemic corticosteroid treatment and orbital radiotherapy during subsequent follow-up were more common in the initially active group, likely because these treatments are more commonly administered to patients with greater disease activity.

Ocular surface disease in TED arises from increased exposure and tear film instability related to eyelid retraction, proptosis, and incomplete blinking [5,6,25]. These changes can promote tear hyperosmolarity and activate inflammatory signaling pathways, including mitogen-activated protein kinase and nuclear factor-κB pathways, thereby increasing the expression of MMP-9 and other inflammatory mediators [7,26,27]. Increased MMP-9 can disrupt the epithelial barrier and further aggravate tear film instability and epithelial inflammation, thereby contributing to a self-perpetuating ocular surface inflammatory cycle [8,27,28]. Accordingly, the higher tear MMP-9 grade, shorter TBUT, and greater CFS observed in this study among patients with active TED may represent interrelated manifestations of the same ocular surface inflammatory process rather than independent findings. Unlike tear MMP-9 grade and TBUT, OSDI, Schirmer I test, and meiboscore do not primarily reflect the current inflammatory status of the ocular surface. Subjective symptoms often show limited agreement with objective ocular surface signs, the Schirmer I test has considerable measurement variability, and meiboscore primarily reflects chronic structural meibomian gland loss rather than active inflammation [19,29,30,31]. These characteristics may explain their weaker associations with TED activity.

Tear MMP-9 grade showed a significant positive correlation with CAS, although the magnitude of the correlation was modest. This positive correlation may reflect partial overlap between the two measures. CAS includes several externally visible inflammatory signs, such as conjunctival redness and chemosis, whereas tear MMP-9 more specifically reflects inflammation at the ocular surface. Accordingly, both measures would be expected to increase in active TED. However, tear MMP-9 may also be influenced by the degree of proptosis, eyelid retraction, incomplete blinking, and tear film instability, all of which vary among patients [5,6,9,25]. These persistent anatomical changes may also account for low-grade MMP-9 positivity in some patients with inactive TED despite minimal orbital inflammatory activity. Consequently, tear MMP-9 grade and CAS are expected to be related but not fully concordant. Moreover, tear MMP-9 is not specific to TED and cannot distinguish TED-associated ocular surface inflammation from primary dry eye disease.

The relationship between tear MMP-9 grade and active TED became more evident when grade 3 was used as the cutoff value. Grade 3 was markedly more frequent in active TED and remained significantly associated with active TED after adjustment for age, sex, and TSHR-Ab level. Although the overall discriminatory performance of tear MMP-9 grade for active TED was modest, grade 3 showed very high specificity together with a high positive predictive value and positive likelihood ratio. This pattern may be explained by the fact that tear MMP-9 reflects ocular surface inflammation secondary to orbital inflammation and anatomical exposure. A grade 3 reaction may require sufficient exposure-related stress and tear film instability to produce substantial ocular surface inflammation and epithelial damage, making such a strong response uncommon in inactive TED and thereby contributing to its high specificity [7,8,27,28]. Conversely, because the extent of proptosis, eyelid retraction, incomplete blinking, and tear film instability varies among patients, not all patients with active TED develop a grade 3 reaction [5,6,25]. This likely contributed to the relatively low sensitivity of grade 3 for identifying active TED. Thus, considering reaction intensity rather than interpreting InflammaDry solely as positive or negative may provide additional information when assessing TED activity, particularly when a strong reaction is present.

This study has several limitations. First, because of the retrospective cross-sectional design, our findings demonstrate an association but cannot establish a causal relationship between tear MMP-9 grade and TED activity. Second, tear MMP-9 was measured at a single time point, preventing evaluation of longitudinal changes during the disease course or in response to treatment. Third, semiquantitative grading was based on visual interpretation and may therefore be subject to interobserver variation, although previous work has reported favorable reliability for standardized grading [11]. Fourth, exposure-related ocular factors, including proptosis, eyelid retraction, incomplete blinking, and tear film instability were not comprehensively evaluated in this retrospective study. Thus, potential confounding by these factors could not be excluded. In addition, a fixed right-eye measurement may not fully correspond to the patient-level CAS in asymmetric TED. Finally, because only 30 patients had a grade 3 result and no independent validation cohort was available, the observed high specificity may be sample-dependent.

## 5. Conclusions

Despite these limitations, our findings demonstrate a graded association between InflammaDry reaction intensity and TED activity, extending previous studies that primarily interpreted InflammaDry dichotomously or evaluated reaction intensity in dry eye disease [10,11,12,21,22,23]. Tear MMP-9 is unlikely to replace CAS but may serve as a complementary biomarker to objectively assess ocular surface inflammation. Prospective longitudinal studies incorporating standardized quantitative assessment are warranted to further establish the clinical utility of tear MMP-9 grading.

## Figures and Tables

**Table 1:** Baseline Characteristics According to Thyroid Eye Disease Activity.

Parameter	Inactive TED (*n* = 188)	Active TED (*n* = 134)	*p* Value
Age (years)	49.87 ± 14.84	51.84 ± 12.55	0.527
Sex (male/female)	58/130	50/84	0.275
Corticosteroid treatment during subsequent follow-up (%)	100 (53.2)	113 (84.3)	<0.001 *
Orbital radiotherapy during subsequent follow-up (%)	10 (5.3)	32 (23.9)	<0.001 *
TSHR-Ab (IU/L)	7.83 ± 11.25	9.39 ± 11.44	0.036 *

Values are presented as mean ± standard deviation or number (percentage). TED = thyroid eye disease; TSHR-Ab = thyroid-stimulating hormone receptor antibody. * *p* < 0.05.

**Table 2:** Ocular Surface Parameters According to Thyroid Eye Disease Activity.

Parameter	Inactive TED (*n* = 188)	Active TED (*n* = 134)	*p* Value
MMP-9 grade	1.26 ± 0.68; 1.5 (1.0–1.5)	1.64 ± 0.98; 1.5 (1.0–2.5)	<0.001 *
OSDI	33.45 ± 23.98	35.94 ± 18.27	0.282
Schirmer test (mm)	11.70 ± 7.77	12.78 ± 8.08	0.163
TBUT (s)	7.86 ± 3.32	6.71 ± 3.03	0.019 *
CFS score	1.26 ± 0.86; 1.0 (1.0–2.0)	1.69 ± 1.11; 2.0 (1.0–3.0)	<0.001 *
Meiboscore	1.17 ± 0.78; 1.0 (1.0–2.0)	1.19 ± 0.65; 1.0 (1.0–1.0)	0.797
LLT (nm)	75.41 ± 23.26	74.96 ± 23.66	0.888

Values are presented as mean ± standard deviation; for MMP-9 grade, CFS score, and meiboscore, median (interquartile range) is additionally provided. TED = thyroid eye disease; MMP-9 = matrix metalloproteinase-9; OSDI = Ocular Surface Disease Index; TBUT = tear break-up time; CFS = corneal fluorescein staining; LLT = lipid layer thickness. * *p* < 0.05.

**Table 3:** Association of Tear MMP-9 Cutoffs with Active Thyroid Eye Disease.

Tear MMP-9 Grade	Inactive TED, *n*	Active TED, *n*	Total, *n*	Active TED Proportion, %	Adjusted OR (95% CI)	*p* Value
Grades 0–1	153	74	227	32.6	1.00	<0.001
Grades 2–3	35	60	95	63.2	3.42 (1.99–5.89)	<0.001
Grades 0–2	184	108	292	37.0	1.00	<0.001
Grades 3	4	26	30	86.7	8.65 (2.86–26.17)	<0.001

Values are presented as numbers, percentages, or adjusted odds ratios (ORs) with 95% confidence intervals (CIs), as appropriate. MMP-9 = matrix metalloproteinase-9; TED = thyroid eye disease; TSHR-Ab = thyroid-stimulating hormone receptor antibody. Models were adjusted for age, sex, and serum TSHR-Ab level.

**Table 4:** Diagnostic Performance of Tear MMP-9 Cutoffs for Identifying Active Thyroid Eye Disease.

Measure	Sensitivity, % (95% CI)	Specificity, % (95% CI)	PPV, % (95% CI)	NPV, % (95% CI)	LR+ (95% CI)	LR− (95% CI)
Grade 3 tear MMP-9	19.4% (13.6–26.9)	97.9% (94.7–99.2)	86.7% (70.3–94.7)	63.0% (57.3–68.3)	9.12 (3.26–25.52)	0.82 (0.76–0.90)
Grades 2–3 tear MMP-9	44.8% (36.6–53.2)	81.4% (75.2–86.3)	63.2% (53.1–72.2)	67.4% (61.1–73.2)	2.41 (1.69–3.42)	0.68 (0.57–0.80)

Values are presented as percentages, likelihood ratios, or 95% confidence intervals (CIs). For the grade 3 cutoff, a grade 3 result is positive and grades 0–2 are negative. For the grades 2–3 cutoff, grades 2–3 are positive and grades 0–1 are negative. MMP-9 = matrix metalloproteinase-9; TED = thyroid eye disease; PPV = positive predictive value; NPV = negative predictive value; LR+ = positive likelihood ratio; LR− = negative likelihood ratio.

## Data Availability

The data generated or analyzed during this study are available from the corresponding authors upon reasonable request.
